# Targeting Notch signaling pathways with natural bioactive compounds: a promising approach against cancer

**DOI:** 10.3389/fphar.2024.1412669

**Published:** 2024-07-18

**Authors:** Jia Yang, Qihui Sun, Xiaoyun Liu, Yong Yang, Rong Rong, Peiyu Yan, Ying Xie

**Affiliations:** ^1^ College of Pharmacy, Shandong University of Traditional Chinese Medicine, Jinan, Shandong, China; ^2^ State Key Laboratory of Quality Research in Chinese Medicine, Macau University of Science and Technology, Taipa, Macao SAR, China; ^3^ State Key Laboratory of Traditional Chinese Medicine Syndrome, The Second Affiliated Hospital of Guangzhou University of Chinese Medicine, Guangzhou, Guangdong, China; ^4^ School of Chinese Materia Medica, Beijing University of Chinese Medicine, Beijing, China; ^5^ Key Laboratory of Traditional Chinese Medicine Classical Theory, Ministry of Education, Shandong University of Traditional Chinese Medicine, Jinan, Shandong, China

**Keywords:** notch signaling, crosstalk, oncogenic pathways, phytochemicals, cancer

## Abstract

Notch signaling pathway is activated abnormally in solid and hematological tumors, which perform essential functions in cell differentiation, survival, proliferation, and angiogenesis. The activation of Notch signaling and communication among Notch and other oncogenic pathways heighten malignancy aggressiveness. Thus, targeting Notch signaling offers opportunities for improved survival and reduced disease incidence. Already, most attention has been given to its role in the cancer cells. Recent research shows that natural bioactive compounds can change signaling molecules that are linked to or interact with the Notch pathways. This suggests that there may be a link between Notch activation and the growth of tumors. Here, we sum up the natural bioactive compounds that possess inhibitory effects on human cancers by impeding the Notch pathway and preventing Notch crosstalk with other oncogenic pathways, which provoke further study of these natural products to derive rational therapeutic regimens for the treatment of cancer and develop novel anticancer drugs. This review revealed Notch as a highly challenging but promising target in oncology.

## Graphical Abstract

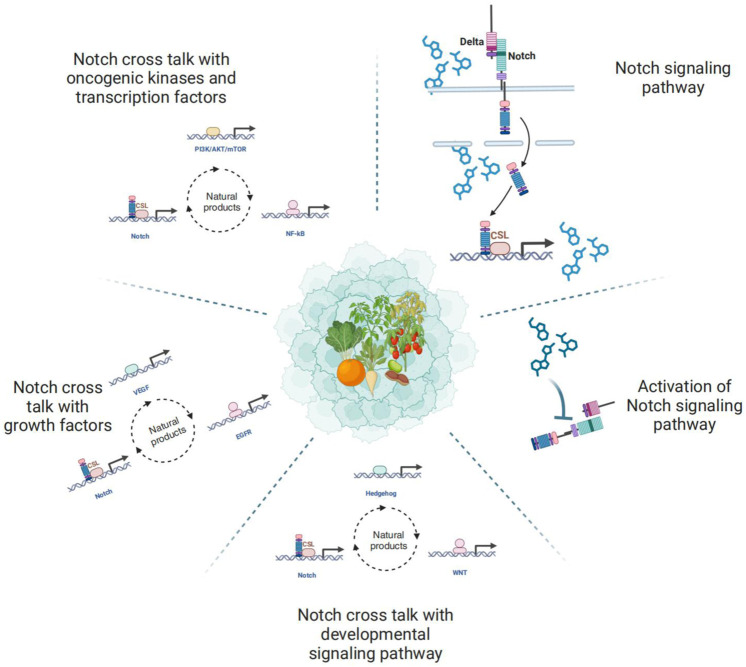

## 1 Introduction

Cancer is a highly destructive illness that poses a significant threat to human wellbeing (Bray et al., 2021). Treatment choices include radiotherapy, chemotherapy, surgery, immunotherapy, and targeted therapy (Ullah et al., 2023). However, there has been limited improvement in the poor prognosis, adverse reactions, drug resistance, and high recurrence rates. Therefore, discovering new therapeutic drugs or targets for cancer is required. Notch, an ideal molecular target, is predominantly expressed in cancerous cells (Espinoza and Miele, 2013).

Notch signaling is a highly conserved pathway that is present in various types of human malignancies, such as breast, colorectal, lung, pancreatic, prostate tumors, and glioblastoma (Majumder et al., 2021). It plays a crucial role in various cellular processes, including cell proliferation, differentiation, and the maintenance of cancer stem cells (Zhou et al., 2022).

Clinical research demonstrates an adverse correlation between the level of Notch expression and patient survival in different kinds of cancer, indicating that increased Notch activation is associated with strengthened cancer aggressiveness (Takebe et al., 2014). Thus, modulating the expression levels of the Notch pathway is a potential therapeutic approach to treating cancer. Several inhibitors targeting the Notch pathway have been developed, but their safety and efficacy are still being evaluated and are not in clinical use (Takebe et al., 2014). Searching for natural inhibitors of Notch with low toxicity and high safety, as well as elucidating their mechanism, could to some extent address the gap of inhibitors that have not yet been utilized in clinical practice. On the other hand, recent work indicates that oncogenic pathways affected by natural bioactive compounds may be linked to or interact with the Notch pathways, suggesting a potential connection between Notch signaling and tumor progression.

In this review, we summarize the role of bioactive natural products in regulating the Notch signaling pathway, particularly focusing on its activation and interaction with other oncogenic signaling pathways in various types of cancer. The aim of this study is to investigate the potential therapeutic applications of natural products targeting Notch in cancer treatment.

## 2 Notch signaling pathway

The Notch signaling pathway includes two primary categories: the canonical pathway and the non-canonical pathway. The canonical Notch signaling pathway plays a crucial role in determining cell fate, including cellular communication, differentiation of embryos and tissues, gene expression, as well as the development of both benign and malignant diseases (Yamamoto et al., 2014).

**FIGURE 1:**
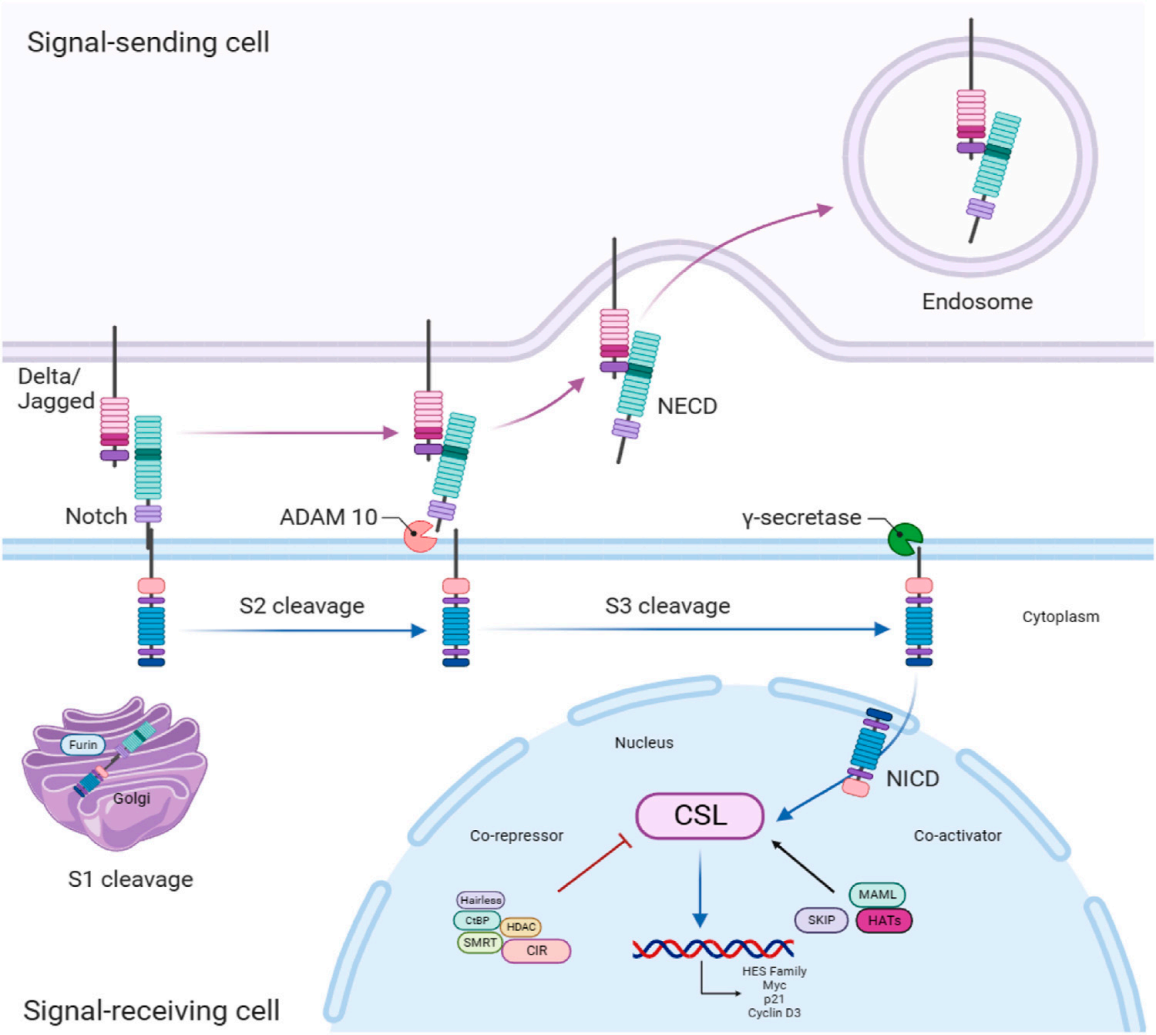
Notch receptors are initially produced as a solitary polypeptide in signal-receiving cells. These receptors are subsequently divided by Furin-like convertase(s) in the trans-Golgi network (S1) and combine to create a heterodimer. During trafficking, this heterodimeric receptor is conveyed to the cellular membrane. In the meantime, Notch ligands in sender cells can attach to Notch receptors in receiver cells. The contact between the receptor and ligand triggers a second cleavage (S2) in the extracellular domain, which is facilitated by the ADAM (A disintegrin and metalloprotease). The Notch extracellular domain (NECD) has a role in the binding of the ligand. Subsequently, a third cleavage (S3) takes place within the transmembrane domain, facilitated by the gamma-secretase function of the presenilin, Nicastrin, Anterior pharynx-defective 1 (APH-1), and Presenilin enhancer 2 (PEN-2) multi-protein complex. Lastly, the intracellular domain of Notch (NICD) is liberated and migrates to the nucleus, where it interacts with the transcription factor CSL (CBF1, Suppressor of Hairless, Lag-1). This connection results in the stimulation of transcription by blocking co-repressors and simultaneously attracting co-activators like mastermind, so facilitating the transcription of Notch target genes. Note: Mastermind-like (MAML); Histone acetyl transferases (HATs); Ski-interacting protein (SKIP); MYC proto-oncogene (Myc); Cold-inducible RNA-binding protein (CIR); Histone deacetylases (HDAC); Nuclear receptor corepressor 2 (SMRT); C-terminal binding protein (CtBP).

The architecture of the pathway comprises Notch receptors (Notch 1–4), Notch receptor ligands, namely, Delta-like (DLL1, -3, and -4) or Serrate-like (commonly known as Jagged 1 and Jagged 2), transcription elements, co-activators, co-repressors, and downstream effectors (Zhou et al., 2022). The activation of the Notch signaling pathway occurs when the Notch ligand combines with the Notch receptor in signal-receiving cells. The Notch receptors undergo three cleavages before translocating into the nucleus in order to regulate the transcription of target genes (Majumder et al., 2021). It is noteworthy that a diverse range of cellular activities can be triggered by identical signaling pathways in distinct circumstances. Considering this, cancer progression activities initiated by Notch and its cross talks are dependent on the context (Guo et al., 2011). The Notch signaling pathway is summarized as depicted in Figure 1.

## 3 Phytochemicals treated cancers via modulating Notch pathway

Various studies have shown that natural bioactive compounds offer substantial protection against cancer by influencing Notch signaling pathways. This includes suppressing Notch pathway activation, inhibiting gamma-secretase expression, Notch transcription complex, and Notch downstream target genes, as well as preventing communication between Notch and other cancer-causing pathways.

### 3.1 Phytochemicals suppressed the Notch signaling pathway’s activation

Notch is initiated by the binding of the extracellular domain of the Notch receptors (Notch1-4) to its ligands (DLL 1, -3, -4, and Jagged1, -2), which are typically present on the surface of neighboring signal-sending cells (Reichrath and Reichrath, 2020). Activation of the Notch signaling pathway controls cell fate and proliferation in both non-cancerous and cancerous diseases (Zhou et al., 2022). The following topics are addressed in our discussion on the regulation of Notch activation using natural products: 1) the modulation of Notch ligands and receptors expression, and 2) the interference with ligand-receptor binding.

Epigallocatechin-3-gallate (EGCG), the main polyphenol in green tea, decreased the expression of Notch receptors including Notch1 (Hossain et al., 2012; Lee et al., 2013; Toden et al., 2016; Wang et al., 2020; Ashry et al., 2022) and Notch2 (Jin et al., 2013) in several cancer models. Notch was activated in the tongue cancer mice and EGCG significantly blocked the activation by decreasing the expression of Notch1, which induced cell apoptosis and inhibited the proliferation (Wei et al., 2022). In addition, EGCG also promotes suppression of Notch1 and cleaved Notch1 in 5-Fluorouracil -resistant cell lines (Toden et al., 2016), suggesting that EGCG may overcome the 5-Fluorouracil resistance by modulating the activation of Notch. However, no studies have indicated that EGCG could regulate Notch ligands.

Resveratrol, isolated from grapes, peanuts, and blueberries, possesses antitumor and antioxidant properties. It has the potential to inhibit cell cycle and growth (Pinchot et al., 2011; Yu X. M. et al., 2013; Zhang et al., 2014), stimulate apoptosis and re-differentiation (Yu XM. et al., 2013), prevent tumor recurrence (Giordano et al., 2023), and impede autophagy (Giordano et al., 2021) in cancer through downregulation of Notch receptors and ligands, including Notch1, Notch2, Notch4, DLL1, DLL4, and Jagged1. These studies indicated that resveratrol could hinder the activation of the Notch pathway through repression of Notch receptors and ligands in several tumors. However, Notch signaling might not play a universally critical role in human medulloblastoma cells given its limited relevance to resveratrol-induced differentiation and apoptosis (Lacerda-Abreu et al., 2021). The combined use of a Notch inhibitor and resveratrol demonstrated greater effectiveness in blocking autophagy, suggesting its potential as a therapeutic intervention for cancer (Giordano et al., 2021).

Curcumin, also termed diferuloylmethane, a bright yellow bioactive compound derived from the plants of the Curcuma longa species. Curcumin decreased the levels of Notch1 (Liu et al., 2014; Chen G. P. et al., 2017; Gupta et al., 2017; Siddappa et al., 2017; Li et al., 2018; Zhdanovskaya et al., 2022), Notch3 (Zhdanovskaya et al., 2022) and Jagged1 (Kiesel and Stan, 2022), leading to cell death mediated by DNA damage and hindering cellular self-renewal, apoptosis (Howells et al., 2011; Liu et al., 2014), motility, migration, and invasion. Importantly, curcumin could upregulate the levels of miR222-3p (Tang and Cao, 2022) and miR-34a (Toden et al., 2018) while downregulating miR-27a (Han et al., 2020), paralogous proteins, and gamma-secretase protein complex (Subramaniam et al., 2012), ultimately leading to the inactivation of Notch1. Curcumin treatment suppressed invasion in human osteosarcoma U2OS cells, and overexpressing Notch1 reversed this effect (Sha et al., 2016), indicating that the antitumor effect of curcumin is mediated through Notch1. However, curcumin had no impact on the expression of Notch1, but it did hinder the DNA-binding capacity of cleaved Notch1 in human prostate DU145 cells (Yang et al., 2017).

Silybin, derived from milk thistle seed, is mainly used in chronic liver disease and is well known for its anti-inflammatory, hepatoprotective, antiviral, neuroprotective, and cardioprotective. Results regarding the efficacy of silybin as a Notch modulator are clear. Notably, the antitumor activity of silybin was weakened by recombinant human Jagged1, suggesting that Jagged1 may be utilized as an anti-tumor target (Zhang et al., 2013; Kim et al., 2014). However, the synergistic effect of Notch1 siRNA *in vitro* or DAPT (C_23_H_26_F_2_N_2_O_4_), a well-known Notch inhibitor) *in vivo*, augmented the anticancer potency of silybin, suggesting that silybin may improve the efficacy of Notch inhibitors.

Activation of Notch signaling has been implicated in pathogenesis of various hematologic tumors including leukemias (Hubmann et al., 2020; Zhang et al., 2022; Skelin et al., 2024) and lymphomas (Alderuccio and Lossos, 2022). For example, in lymphopoiesis, the Notch1 activation may be involved in the correct differentiation of T and B cells (Okatani et al., 2024). In chronic lymphocytic leukaemia, Notch1 may represent a potential druggable target (Pozzo et al., 2022), suggesting that phytochemicals, such as EGCG and curcumin, may be beneficial in the treatment of chronic lymphocytic leukemia (CLL). Suppressing the activity of Notch1 or Notch2 resulted in an enhanced response of CLL cells to fludarabine (Del Papa et al., 2019) or venetoclax (Fiorcari et al., 2022), respectively, indicating that natural compounds with the ability to decrease Notch activation may be able to overcome resistance in CLL. It is crucial to highlight that in T cell acute lymphoblastic leukemia (T-ALL), the Notch1 activating mutations typically occurs later in a sequence of genomic damages, suggesting that Notch1-based therapies may impede the efficacy targeted Notch1 therapy (De Bie et al., 2018).

**FIGURE 2:**
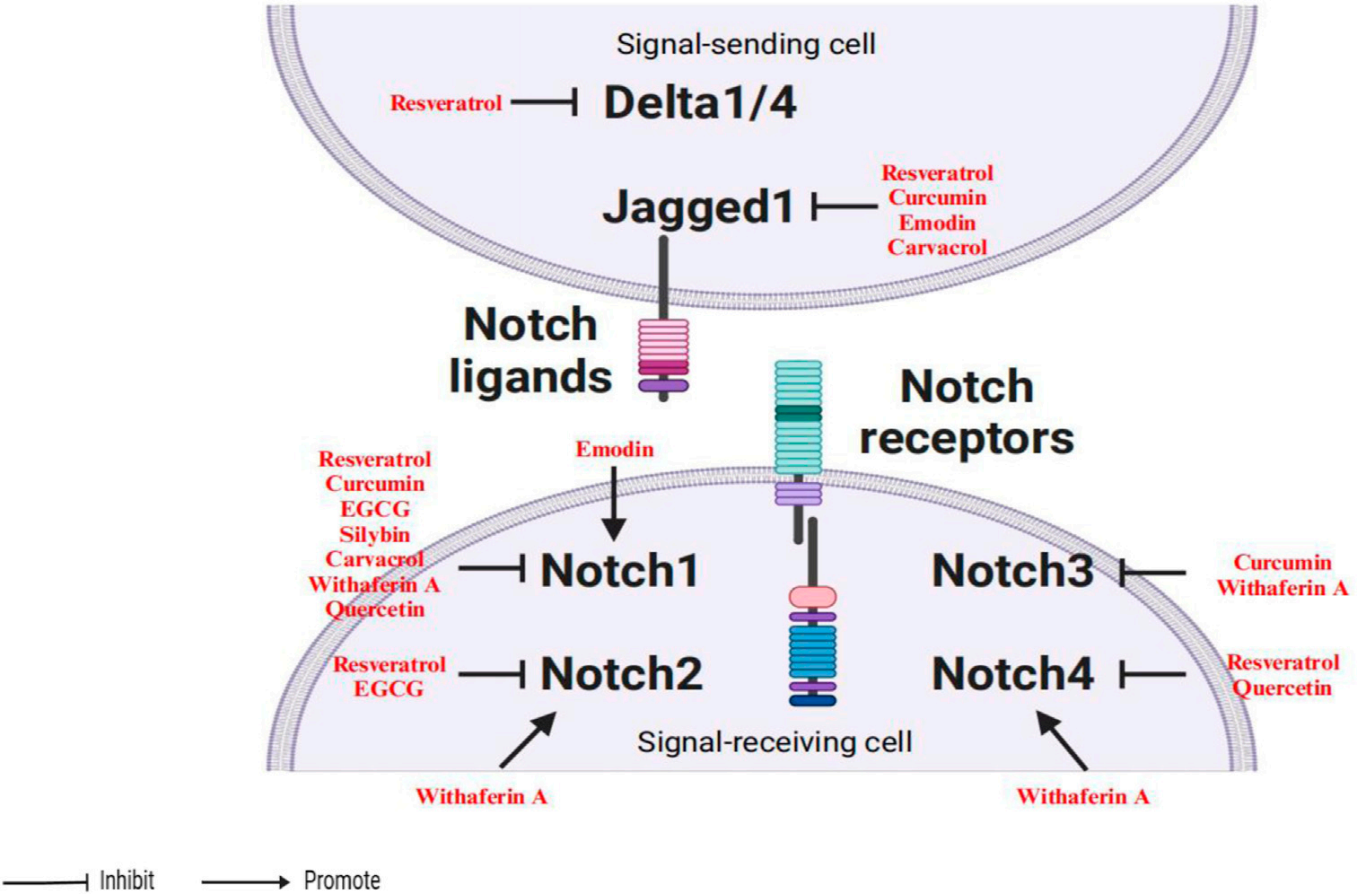
Phytochemicals suppress the activation of the Notch signaling pathway.

Other agents could also inhibit Notch pathway activation by controlling the expression of both Notch receptor and ligand, such as emodin (Deng et al., 2015), carvacrol (Khan et al., 2019), withaferin A (Lee et al., 2012) and quercetin (Okuhashi et al., 2011; Peiffer et al., 2020). In summary, several natural bioactive compounds suppress Notch activation to downregulate cancer aggressiveness as shown in Figure 2.

### 3.2 Phytochemicals modulate the Notch signaling pathway

Phytochemicals may play an anti-tumor role by modulating other components of the Notch pathway, such as the gamma secretase complex, the Notch transcription complex and Notch downstream target genes.

#### 3.2.1 Gamma-secretase inhibitors

The gamma-secretase complex, which consists of presenilin, Nicastrin, APH-1, and PEN-2 proteins, plays a crucial role in producing the NICD. Inhibition of secretase activity has shown significant antitumor efficacy in cancers, including lung cancer, colorectal cancer, melanoma, and ovarian cancer (Takebe et al., 2014). Several gamma-secretase inhibitors (GSIs) have advanced from preclinical testing to early clinical stages and observed antitumor activity (Mccaw et al., 2021). Unfortunately, clinical trials had to be stopped due to serious adverse events, such as gastrointestinal toxicities, skin disorders, and diarrhea, making the use of inhibitors less attractive for Notch blockade to arrival antitumor effects. Therefore, it is necessary to explore new strategies via inhibiting gamma-secretases.

Cucurbitacin B and I, decrease the expression of Notch receptors and ligands, repressing the activity of gamma-secretases, suppressing NICD production, binding to Notch1, subsequently repressing downstream genes of Notch, inhibiting tumor growth in colon cancer (Dandawate et al., 2020; Mccaw et al., 2021). Celastrol and triptolide both reduced the expression of Notch1 and its downstream target proteins, hence regulating the renewal of stem cells in triple negative breast cancer (Ramamoorthy et al., 2021). Additionally, quercetin also decreases the expression of all five proteins (presenilin1, presenilin2, Nicastrin, APH1, PEN2) of the gamma-secretase complex in colorectal cancer (Li et al., 2020). Combining quercetin with a gamma-secretase inhibitor (Okuhashi et al., 2011) or ionizing radiation (Liao et al., 2023) could enhance the overall anti-tumor efficacy, implying that quercetin may be used to reduce the toxicity of GSIs.

#### 3.2.2 Notch transcription complex inhibitors

The interaction between NICD and CSL plays a crucial role in determining the activation or deactivation of downstream genes in the nucleus within the Notch signaling pathway. Phytochemicals inhibit the binding of CSL and NICD, as well as the expression of co-activators, while boosting the expression of co-repressors, resulting in antitumor effects. For example, silybin suppresses the Notch signaling system by activating the apoptotic pathway, leading to the inhibition of NICD activity in human cancer cells. Silybin reduces the expression of intracellular domain of Notch1 (N1ICD) and RBPJ (recombination signal-binding protein of immunoglobulin kappa J region) activity in hepatocellular carcinoma with a CSL-dependent manner (Zhang et al., 2013), suggesting that blocking CSL and N1ICD interactions is a good idea in tumor therapy. Resveratrol alters the DNA methylation patterns in human breast cancer cells and inhibits the cancer-causing Notch signaling by controlling the co-activator (MAML2) transcriptional activity through epigenetic processes (Lubecka et al., 2016). Similarly, resveratrol also inhibits the co-activator protein ASCL1 in carcinoid tumors (Pinchot et al., 2011). Resveratrol could enhance the recruitment of the co-repressor (HDAC1) in human glioma cells (Yang et al., 2016). Therefore, it is crucial to limit the activation of co-activators or promote the activation of co-repressors in tumor cells to avoid the development of cancer.

#### 3.2.3 Notch downstream target genes inhibitors

The ultimate stage in Notch signaling is the transcription of downstream target genes. The Hes and Hey protein families are the most extensively studied targets of Notch (Guo et al., 2011). Hes1 has been demonstrated to promote the activation of the Notch signaling pathway, thereby promoting the proliferation and inhibiting the apoptosis. Natural products, such as oleandrin and cowanin, have been shown to depress the expression of Hes1/5, thus controlling the progression of T-ALL (Arai et al., 2018a; Arai et al., 2018b). Several phytochemicals have shown potential in treating cancer by influencing the activity of specific genes downstream of the Notch signaling pathway, as indicated in Table 1.

**TABLE 1:** Phytochemicals on Notch downstream target genes in different types of cancer.

Phytochemical	Cancer type	Hes-1	Hes-5	Hey-1	Hey-2	References
Curcumin	Colorectal Carcinoma	↓				Chen et al. (2017a)
	Pancreatic Cancer	↓				Wang et al. (2006a)
	Melanoma Cancer	↓				Tang and Cao (2022)
	Cholangiocarcinoma	↓				Koprowski et al. (2015)
	Esophageal Cancer	↓				Subramaniam et al. (2012)
Honokiol	Colon Cancer	↓				Ponnurangam et al. (2012)
Curcumin	Colon Cancer	↓				Bounaama et al. (2012)
EGCG	T-ALL	↓				Wang et al. (2020)
	Tongue Cancer	↓				Wei et al. (2022)
3,6-Dihydroxyflavone	Breast Cancer	↓				Chen et al. (2016a)
Resveratrol	Osteosarcoma	↓	↓	↓	↓	Liu et al. (2016)
Triptolide	Triple-Negative Breast Cancer	↓		↓		Ramamoorthy et al. (2021)
Silybin	Hepatocellular Carcinoma	↓				Zhang et al. (2013)
Withaferin A	Breast Cancer	↓		↓		Lee et al. (2012)
Carvacrol	Prostate Cancer					Khan et al. (2019)
Retinoic Acid	Glioblastoma			↓		Ying et al. (2011)
Oleandrin	T-ALL	↓	↓			Arai et al. (2018a)
Cowanin	T-ALL	↓	↓			Arai et al. (2018b)

↓ showed decreased expression.

### 3.3 Crosstalk between Notch and other oncogenic pathways

In human cancers, Notch signaling plays multiple roles in different tissues, acting as both a tumor suppressor and a tumor promoter. Activation of the Notch signaling pathway upregulates multiple factors, which subsequently transmit bidirectional signals among cancerous, stromal, and endothelial cells (Rizzo et al., 2008; Yamamoto et al., 2014). Thus, it is expected that Notch signaling intersects with multiple oncogenic signaling pathways, including Wnt and Hedgehog signaling, growth factors like epidermal growth factor receptor (EGFR), transforming growth factor type beta (TGF-β), and vascular endothelial growth factor (VEGF) oncogenic kinases, and transcription factors like Nuclear factor kappa-B (NF-κB), PI3K (phosphatidylinositol 3-kinase)/AKT (protein kinase B)/mTOR (mammalian target of rapamycin) (Guo et al., 2011). The crosstalk between Notch and other oncogenic signaling pathways is crucial for various cellular processes such as cell proliferation, migration, invasion, metastasis, angiogenesis, and the self-renewal of cancer stem cells, suggesting that Notch signaling could be a promising approach for cancer therapy. Here, we have attempted to summarize how natural products regulate the complexity of cellular responses due to the crosstalk between signaling pathways.

#### 3.3.1 Interacting with developmental signaling pathways

**FIGURE 3:**
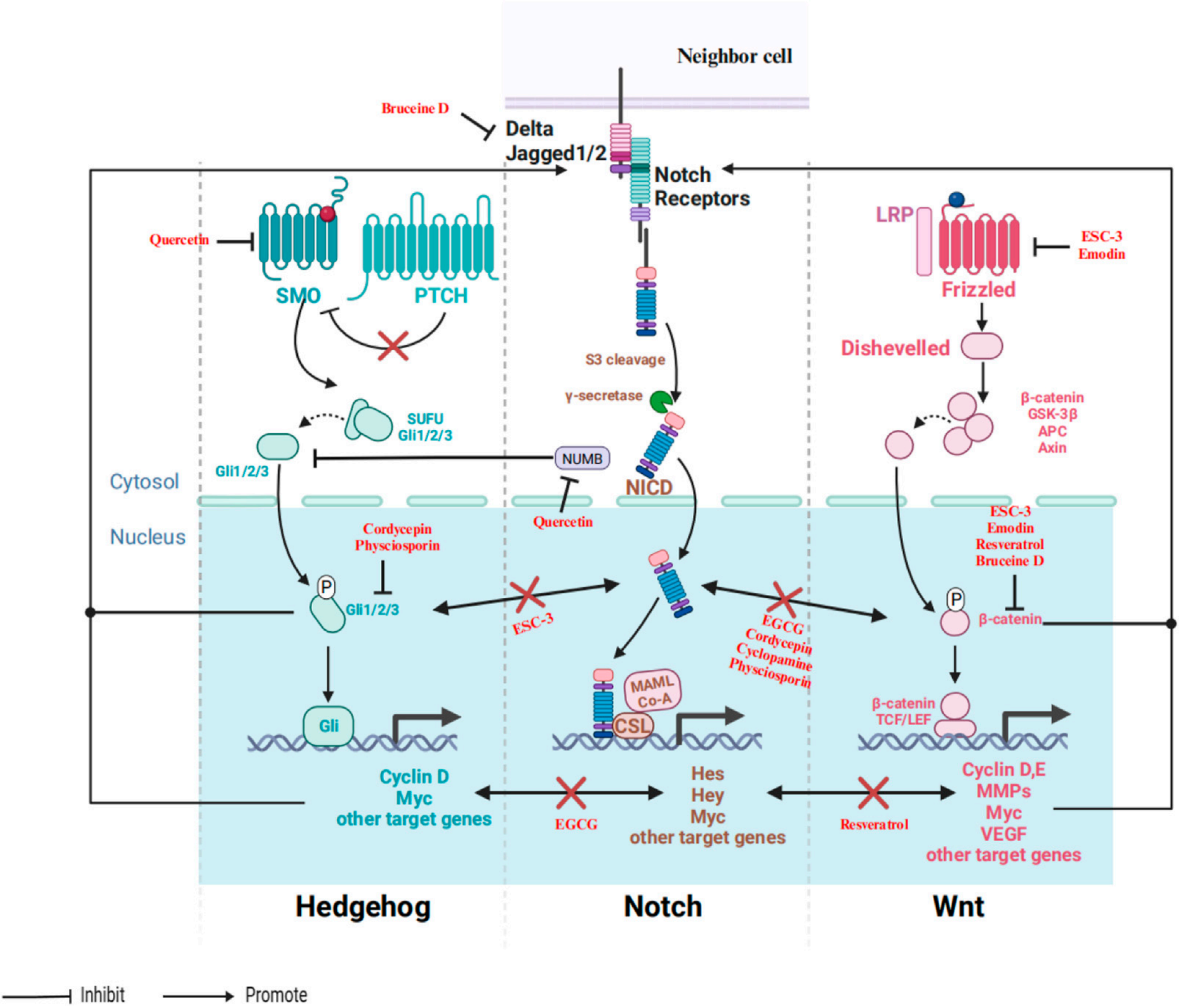
An illustrative depiction of the phytochemicals could influence the interaction between the Notch and Wnt/Hedgehog signaling pathways. In the figures provided, the term “promote” signifies that the chemical(s) could enhance the expression or activity of the specific protein. Conversely, the term “inhibit” suggests that the compound(s) can decrease the expression of the protein or hinder its activity.

During embryogenesis, the Notch pathway interacts with other developmental pathways, including Wnt and Hedgehog signaling pathways, which operate in coordination across various types of cancer (Guo et al., 2011). Interaction among Notch, Hedgehog, and Wnt signaling pathways involved in regulating self-renewal, proliferation, and differentiation, ensuring correct organogenesis (Cerdan and Bhatia, 2010). Therefore, comprehensive knowledge of the interactive functions of these pathways in cancer may provide novel options for cancer treatment as shown in Figure 3.

The Wnt signaling pathway is a major developmental pathway that determines cell proliferation, differentiation, tissue homeostasis, and epithelial-mesenchymal interactions during embryogenesis (Arai et al., 2018b; Manoranjan et al., 2020). The pathway comprises two major categories: the canonical Wnt pathway, which involves key elements such as Wnt, β-catenin, and TCF/LEF (T-cell factor/lymphoid enhancer factor) transcription factors, and the non-canonical Wnt-calcium pathway, which regulates intracellular calcium levels and cytoskeleton of cells (Vlashi et al., 2023). Therefore, the suppression of Wnt activity has received extensive attention in the study of cancer cells, presenting an opportunity for cancer treatment (Olson et al., 2006; Seifert and Mlodzik, 2007).

A recent study revealed the expression of Notch ligands inhibited the transformation of human mammary epithelial cells induced by Wnt1, suggesting that the involvement of Notch-Wnt communication in breast tumorigenesis (Ayyanan et al., 2006). ESC-3, a novel cytotoxic compound, induces apoptosis via inhibiting both Notch and Wnt/β-catenin pathways, resulting in a reduction in tumor growth in an ovarian cancer xenograft model (Fu et al., 2017). Emodin also hinders the Wnt pathway by reducing the level of active β-catenin in human glioma stem cells (Kim et al., 2018).

β-catenin has been shown to activate Notch signaling by upregulating the expression of the Notch ligand Jagged1 (Rodilla et al., 2009; Kode et al., 2014). The efficacy of Bruceine D, which is derived from the Chinese herb *Brucea javanica* (L.) Merr, to inhibit Jagged1 was found to be synergistic following β-catenin knockdown and reversed following overexpression (Cheng et al., 2017). However, the levels of β-catenin remained unchanged in Jagged1 knockdown cells (Cheng et al., 2017). It is reasonable to assume that the tumorigenesis mediated by β-catenin could be blocked by Notch inhibitors, such as resveratrol, 8,12-dimethoxysanguinarine, and emodin. Conversely, the activated Wnt signal boosts the accumulation of β-catenin in the nucleus. Subsequently, specific target genes undergo transcriptional activation, leading to the development of cancer (MacDonald et al., 2009). Resveratrol inhibited the proliferation of ovarian cancer cells by reducing the expression of β-catenin and Hes1, suggesting the simultaneous suppression of the biological functions of Notch and Wnt signaling (Zhong et al., 2015). In summary, the regulation of β-catenin is vital in the crosstalk between Wnt and Notch signaling.

The Hedgehog is a developmental signaling pathway that plays an essential role in embryogenesis, tissue polarity, tissue regeneration, and carcinogenesis (Lum and Beachy, 2004; Li et al., 2012). The Hedgehog pathway consists of several key components, namely, Hedgehog, Patched receptor (PTCH), Smoothened (SMO), cytosolic intermediates like Suppressor of Fused (SUFU) and Costal 2 (COS2), as well as inhibitors such as Protein Kinase A (PKA) and Supernumerary Limbs (SLIMB). Additionally, the pathway involves the transcription factors Cubitus interruptus/Zinc Finger protein -Glioma-associated oncogene homolog1 (Ci/Gli) (Cortes et al., 2019). Hedgehog has three ligands, Sonic hedgehog, which is critical for neuronal development, Indian hedgehog, which is critical for skeletal development, and Desert hedgehog, which is critical for gonadal development (Lee et al., 2016).

According to the literature, Hedgehog is controlled by the Notch signaling pathway, mainly through the downstream effector of Notch that controls the transport of Hedgehog component and Gli levels (Xia et al., 2022). Consequently, Notch target genes play a central role in controlling Gli gene transcription (Jacobs and Huang, 2019). Physciosporin, derived from *P. granulata*, decreased the transcriptional activity of the Gli, Hes1 and CSL (Yang et al., 2019). Additionally, it markedly reduced the formation of spheroids in human CSC221 cells that overexpressed Gli1/2 or Delta EN1 (a membrane-bound and S2-cleaved type of human Notch1). Nevertheless, it did not decrease the formation of spheroids in cells that overexpressed both Gli1/2 and Delta EN1, proposing that physciosporin can downregulate the cancer stemness of human colon cells by controlling the Sonic Hedgehog and Notch signaling pathways (Yang et al., 2019). Additionally, NUMB endocytic adaptor protein (NUMB) negatively regulates Notch signaling by binding directly to NICD, preventing NICD from initiating gene transcription (Flores et al., 2014). NUMB is also a suppressor of Hedgehog signaling and specifically targets Gli1 for ubiquitination through the action of Itch (Di Marcotullio et al., 2006). Quercetin decreases tumor growth by inhibiting the activation of NUMB and Hedgehog signaling pathway (Salama et al., 2019), suggesting that NUMB has potential as a biomarker for cancer.

Additionally, modulation of the Notch pathway by the Hedgehog signaling pathway, mainly through inhibition of Hedgehog downstream effectors, such as Gli proteins and Hes1 (Wall et al., 2009; Dave et al., 2011). Hedgehog signaling may induce Hes1 expression in Hep2 cells (human), however, this effect can be reversed by EGCG, which acts as an inhibitor for NICD (Ashry et al., 2022). Therefore, the Hedgehog signaling pathway may have an impact on the activity of NICD, leading to the promotion of Hes1 production. Cordycepin, also known as 3-deoxyadenosine, encompasses anti-inflammatory, antioxidant, and anti-cancer activities (Tuli et al., 2014). Cordycepin suppressed the transcriptional activity of Gli, which in turn reduced the expression of Notch1, Notch3, Jagged1 and Hes1 in human triple-negative breast cancer cells (MDA-MB-231 cell line) (Liu et al., 2020). Notably, knocking out of Gli obstructed cordycepin-induced influences on the apoptotic, EMT, and Notch pathways, demonstrating that the regulation of Notch by cordycepin is dependent on Gli in breast cancer (Liu et al., 2020). Evidence indicates that the Notch ligand, Jagged 2 is induced by Hedgehog signaling during carcinogenesis (Katoh, 2007). For example, cyclopamine inhibits the expression of Notch1, Notch2, Notch3, Jagged2, and DLL1 correlated with the downregulation of the sonic hedgehog odontogenic keratocytes (Ren et al., 2012). In summary, the regulation of processing and nuclear translocating of Gli or NUMB is vital in the crosstalk between Hedgehog and Notch signaling.

#### 3.3.2 Interacting with growth factors

**FIGURE 4:**
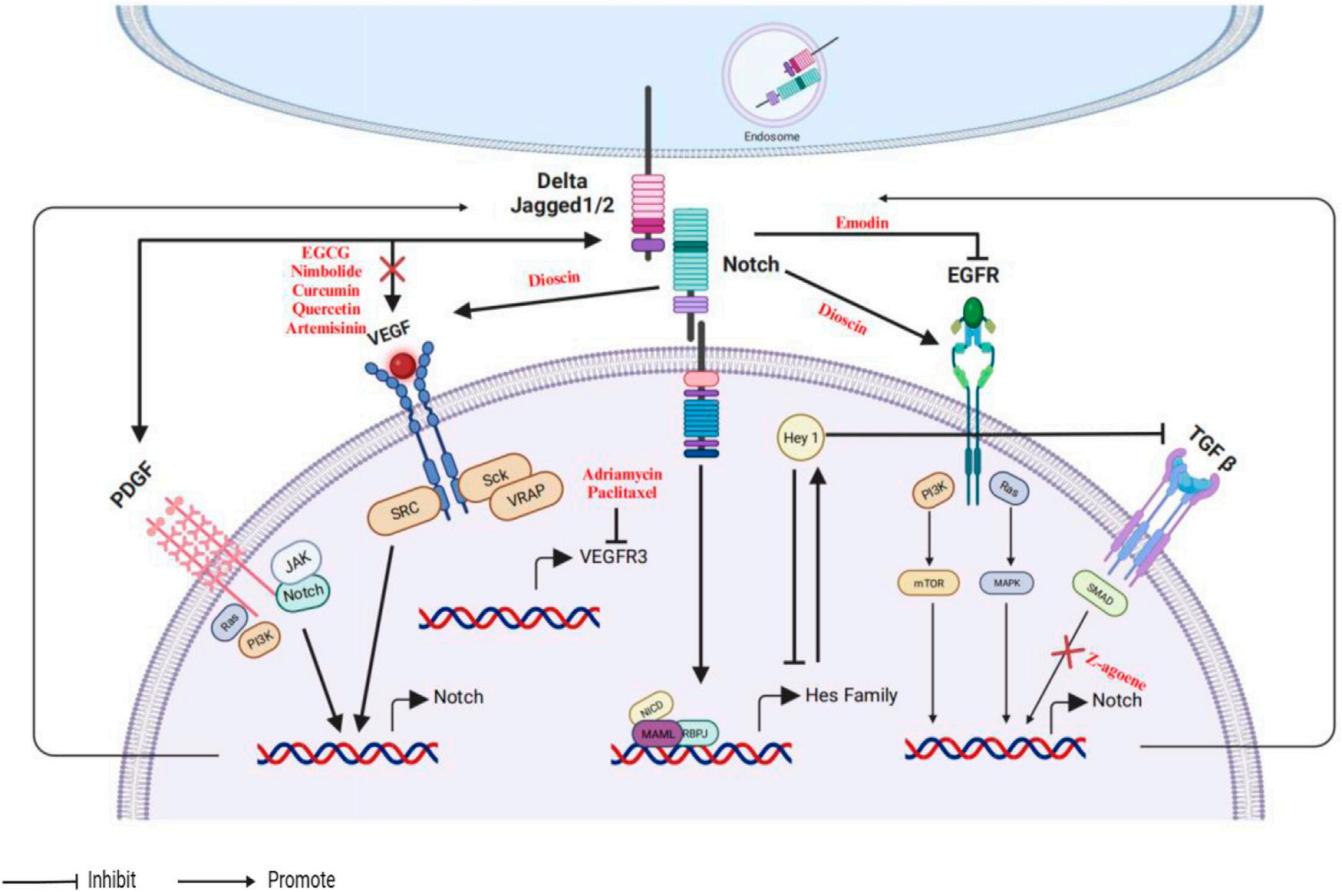
An illustrative depiction of the phytochemicals could influence the interaction between the Notch and EGFR/PDGF/TGF-β/VEGF signaling pathways.

Numerous growth factors exert a variety of actions in cancers. For example, the oncogene gene mutations, amplification or overexpression of HER2/Neu (ErbB2), platelet-derived growth factor (PDGF), EGFR and TGF-β (Wang et al., 2010). Growth factors pathways are associated with the progression of cancers, including proliferation, invasion, and tumor growth. Not surprisingly, the interaction between growth factors and the Notch signaling pathway is frequently observed in several cancers. A diagram illustrating the interplay among different genes of Notch, EGFR, PDGF, TGF-β, and VEGF is presented in Figure 4.

The role of the Notch-EGFR crosstalk has been discovered in various types of cancer, including breast (Baker et al., 2014), lung (Konishi et al., 2007), brain (Purow et al., 2008) cancer. Recently, researchers have continued to explain crosstalk between Notch and EGFR to dissect the mechanisms for a better understanding of how cancer cells apply the Notch pathway to counteract the inhibitory effects of EGFR targeting. Notch signaling could regulate EGFR activity via regulating the nuclear translocation of the Notch1 intracellular domain. For example, dioscin markedly enhanced the expression of Notch1 and Jagged1, and enhanced the activity of gamma-secretase, leading to EGFR, VEGF, and Notch-dependent target genes (Xu et al., 2018). Conversely, the decrease in activity of Notch3 greatly inhibited the growth and stimulated the programmed cell death of the human ErbB2-negative tumor cell lines (Yamaguchi et al., 2008). On the other hand, EGFR signaling may also regulate the Notch pathway. Emodin inhibited the growth of human glioma stem cells through the induction of proteasomal degradation of EGFR/EGFRvIII, which subsequently hindered the activation of stemness signaling pathways, specifically the Notch pathway (Kim et al., 2018).

PDGF, which is produced in carcinomas, primarily affects the non-epithelial tumor stroma, and stimulates angiogenesis (Papadopoulos and Lennartsson, 2018; Li et al., 2022). The available literature indicates that the PDGF receptor is a novel Notch target gene (Wu et al., 2021; Vimalraj, 2022). PDGF-D is a vital factor in the aggressiveness of breast tumors, which is causally associated with the activation of Notch1 (Ahmad et al., 2011). Furthermore, the downregulation of PDGF-D results in the inactivation of the Notch1/Twist1 axis, potentially reversing epithelial-mesenchymal transition and inhibiting the progression of colorectal cancer (Chen J. H. et al., 2017). Unfortunately, there are currently no documented findings on the identification of natural compounds that modulate the communication between PDGF-D and Notch signaling in cancer. Nevertheless, studies have demonstrated that lycopene, a naturally occurring carotenoid found in tomatoes, can effectively suppress the growth of certain cancer cells (Wu et al., 2007). Additionally, it has been observed that lycopene directly binds to PDGF-BB, indicating that its ability to inhibit PDGF may play a role in its anti-tumor properties (Wu et al., 2007).

TGF-β, a multifunctional cytokine, plays a key role in the cancer pathogenesis involved in cell growth, differentiation, adhesion, and apoptosis (Gu and Feng, 2018). The diverse roles of TGF-β in cancer are influenced by its interactions with several signaling pathways, such as Hedgehog, Wnt, PI3K/AKT, Notch, and RAS-ERK (extracellular signal-regulated kinase). Heyl, a downstream effector of the Notch signaling pathway, inhibits the action of TGF-β by interacting with activated Smads (Han et al., 2014), providing novel strategies and perspectives for treating breast cancer. Moreover, Z-ajoene, which is derived from garlic, has demonstrated a variety of biological activities, such as anti-proliferative, antioxidant, and antitumor (Naznin et al., 2010; Jung et al., 2014). Z-ajoene reduced levels of Notch target genes, and TGF-β is the key mediator of the Z-ajoene effect on glioblastoma multiforme cancer stem cells (Jung et al., 2014). Furthermore, the TGF-β pathway is critical in maintaining stem cell properties in cancer cells (Gurska et al., 2019).

VEGF, a significant angiogenic factor in both normal and abnormal blood vessel formation, is frequently overexpressed in human tumors, leading to the malignancy progression across several tumors and low survival rates of patients (Yang and Cao, 2022; Ghalehbandi et al., 2023). Both the VEGF and Notch signaling pathways have been indicated to be key regulators in developmental and pathological angiogenesis, including in cancers (Li and Harris, 2009; Blanco and Gerhardt, 2013). VEGF modulates the expression of Notch signaling components, specifically by upregulating the expression of DLL4 and Notch receptors, which then promotes the activation of Notch signaling (Patel et al., 2005; Hainaud et al., 2006; Ridgway et al., 2006). The combination of survivin shRNA and EGCG markedly decreased angiogenic (VEGF and b-FGF) factors and then inhibited Notch1 expression in neuroblastoma, reasonably inferring that blocking VEGF may enhance the sensitivity of tumors to anti-Notch therapy (Hossain et al., 2012). Nimbolide, obtained from neem leaves and flowers, inhibited the activity of MMP and blocked Notch and VEGF signaling pathways by targeting miR-21 (Kowshik et al., 2017). Curcumin also could decrease the tumor weight and size via downregulating the expression of Notch, HIF-1, VEGF, and NF-κB (Li et al., 2018).

Although VEGF induces Notch signaling, expression of VEGF ligands and receptors appear to be also regulated by Notch signaling. For example, quercetin decreased Notch1 expression and then suppressed the expression of angiogenesis-associated proteins hypoxia-inducible factor alpha (HIF1α) and VEGF in histiocytic lymphoma (Chen X. et al., 2016). In breast cancer, artemisinin, an anti-malarial active compound derived from the sweet wormwood plant, downregulates the expression of Notch1, DLL4, and Jagged1, reducing VEGF and HIF-1α levels (Dong et al., 2020). Providing a feedback mechanism, another study has found that the inhibition of Notch signaling or the suppression of Notch4/DLL3 leads to a decrease in endothelial markers and the function of tumor-derived endothelial cells following treatment with adriamycin or paclitaxel via VEGF receptor (Zhang et al., 2016). These data provide new prospects for the antiangiogenic therapy of human cancer.

#### 3.3.3 Interacting with oncogenic kinases and transcription factors

The NF-κB pathway comprises two major categories: the canonical IKK pathway which is dependent on IκB proteins through an individual kinase signalosome multiprotein complex, while the non-canonical pathway is triggered by ligands like CD40L and lymphotoxin (Dolcet et al., 2005). NF-κB activation is evident in several cancer types and is also associated with the initiation of tumor angiogenesis (Sarkar and Li, 2008; Prasad et al., 2010). Many publications have described how natural products can regulate the NF-κB pathway through Notch, and *vice versa*, by various context-dependent mechanisms (Rajasinghe and Gupta, 2017; Ferrandino et al., 2018; Hossain et al., 2018; Grazioli et al., 2020). Firstly, evidence shows that Notch serves as a crucial regulator of NF-κB at an upstream level and regulates NF-κB pathway members (Mishra et al., 2021). RBPJ functions as a potent transcriptional inhibitor of p100/p52, but its inhibitory effects can be reversed by activated Notch1, implying that p100/p52 is regulated by Notch signaling and can be considered a target gene of Notch (Oswald et al., 1998). Triptonide, a natural small molecule extracted from Tripterygium *wilfordii* Hook *F*, efficiently inhibits tumor growth and metastasis via decreasing the levels of Notch1 downstream proteins RBPJ, IKK alpha, IKK beta, reducing p52 phosphorylation (Xiang et al., 2020). Notch1 co-localizes with IKK at NF-κB-responsive promoter sites, enhancing IκB kinase activity and thereby maintaining NF-κB activity (Song et al., 2008). And inactivation of NF-κB DNA-binding activity by genistein was partly impeded by the overexpression of Notch1 in cDNA-transfected BxPC-3 cells (Wang et al., 2006b). Genistein also inhibits tumor growth in pancreatic (Wang et al., 2006b), colon (Zhou et al., 2017), and triple-negative breast cancers (Pan et al., 2012) by inhibiting NF-κB activity via the Notch1 pathway. Curcumin downregulates Notch1 expression, blocks Notch1 activation, and inactivates NF-κB DNA-binding activity (Gupta et al., 2017).

Secondly, NF-κB subunits regulate the transcription of Notch family members. This finding is supported by Wang et al. (2001) which revealed that the N-terminal region of Notch1 NICD specifically associated with the p50 subunit and prevented it from binding to DNA in NTera-2 cells. Lastly, not all cancer cells exhibit a close association between the activation of the NF-κB pathway and the Notch pathway. For example, trichostatin A, derived from *streptomyces*, caused gastric cancer cells growth arrest and apoptosis by controlling NF-κB and p21^WAF1/CIP1^, without the involvement of the Notch pathway (Yao et al., 2012). Another is berberine, which inhibits the Notch, MAPK, and NF-κB signaling pathways by regulating circRNA (Wang et al., 2021). Sentrin-specific protease-2 (SENP2) could act as a tumor suppressor in CLL cells via suppressing the Notch and NF-κB signaling pathways (Chen et al., 2019). Nevertheless, there is currently no natural products that regulates the activity of SENP2 in the therapy of hematologic tumors. Betanidin, obtained from red beets, could directly bind to SENP2 and may be used as a treatment for CLL cells (Taghvaei et al., 2022). These results suggest novel understandings of the crosstalk between Notch and NF-κB.

Thirdly, in cancer biology, as mentioned above, the Notch and NF-κB pathways promote cancer progression by regulating each other’s activities. The Notch-NF-κB network are typically involved in regulation of inflammatory disease (Liubomirski and Ben-Baruch, 2020). Nevertheless, the growth of tumor and its reaction to treatment are controlled by inflammation, which can either stimulate or inhibit the advancement of tumors, potentially leading to contrasting impacts on the effectiveness of therapy (Zhao et al., 2021). Thus, an optimal approach is evidently necessary to address the interaction between the Notch and NF-κB pathways to devise innovative treatments for the treatment of inflammatory illnesses. Utilizing small molecule inhibitors that specifically target NF-κB, which is the downstream of Notch, and not necessarily the Notch blockers, may serve as an effective therapeutic method to disturb the interplay and reduce inflammation. Many of the transformational events in cancers are the consequence of amplified signaling within the PI3K/AKT pathway (Hynes and Stoelzle, 2009; Dillon and Muller, 2010). The PI3K/AKT/mTOR pathway is crucial in regulating multiple cellular activities, such as growth, proliferation, metabolism, motility, migration, invasion, angiogenesis, survival, and autophagy (McAuliffe et al., 2010). And mTOR is a crucial protein kinase that frequently acts as a downstream effector of the PI3K/AKT signaling pathway in various cancer cell types (Carino et al., 2008). mTOR can also phosphorylate AKT (Saxton and Sabatini, 2017). The PI3K/AKT pathway plays a crucial role in promoting EMT during the development of cancer (Sabbah et al., 2008). Crosstalk between PI3K/AKT and Notch pathways has been described in prostate cancer (Wang et al., 2011a), T-ALL (Cecchinato et al., 2007), colon cancer (Koduru et al., 2009), brain cancer (Xiang et al., 2020) as well as breast cancer (Cao et al., 2018). Kim et al. (2014) reported that overexpression of Notch1 reversed the suppression of ERK and AKT phosphorylation caused by silybin. Additional reports also demonstrated that inhibit Notch1 activation by Withaferin A, which resulted in the downregulation of pAKT and Bcl-2 expression (Koduru et al., 2009). Kim et al. (2019) found that induces cell death through ROS-dependent downregulation of Notch1, which negatively controlled the expression of PTEN (Phosphatase and tensin homolog) and AKT signaling. Curcumin, an inhibitor of Notch, increases PTEN expression and decreases AKT phosphorylation in chronic myelogenous leukemia (Chen et al., 2015). Notch inhibits the dephosphorylation of PI3K/AKT by blocking the activation of PP2A (Protein phosphatase 2) and PTEN, leading to the promotion of cancer’s malignant progression (Li et al., 2016). The downregulation of Notch1 activity by okadaic acid, a PP2A inhibitor, decreases low cell invasion in breast cancer via inactivating the AKT, mTOR, and NF-κB signaling pathways (Li et al., 2016). Moreover, through the downregulation of Notch1/PTEN/AKT signaling, resveratrol causes the demise of ovarian cancer cells (Kim et al., 2019).

**FIGURE 5:**
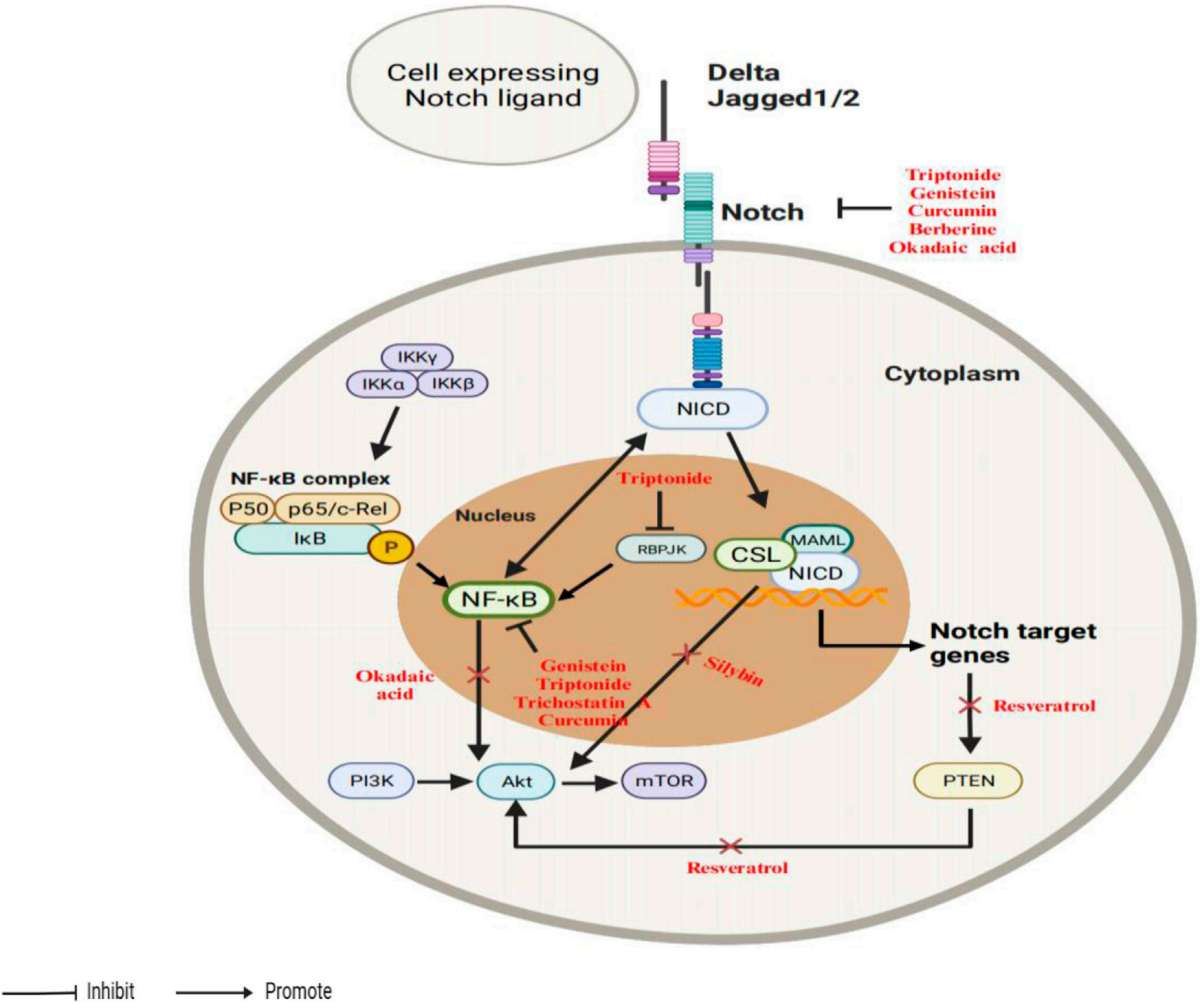
An illustrative depiction of the phytochemicals could influence the interaction between the Notch and PI3K/AKT/mTOR signaling pathways.

AKT serves as a regulator of Notch signaling. On the one hand, Notch1 downregulation is linked to AKT in the induction of cell growth inhibition and death by genistein in prostate cancer (Wang et al., 2011a). On the other hand, resveratrol suppresses Notch signaling in T-ALL cells, resulting in a reduction in AKT activity and modulating the operation of interacting signaling systems (Cecchinato et al., 2007). Therefore, strategies aimed at modulation of Notch-PI3K/AKT/mTOR signaling have the potential to enable therapeutic intervention. A schematic diagram depicting the interactions among the Notch, PI3K/AKT/mTOR, and NF-κB as shown in Figure 5.

#### 3.3.4 Others

In addition, many connections between the Notch pathway and other signaling pathways such as DXX6, ERK, and Hippo signaling may also contribute to the complexity of tumor angiogenesis and the challenge of Notch as a promising target.

The regulation of Notch-related pathways is also irregular with bioactive natural compounds. Regarding breast cancer, the repression of Notch4 protein and its downstream targets by quercetin appears to be independent of death-associated factor 6, whereas genistein is known to repress Notch signaling solely through the presence of death-associated factor 6 (Peiffer et al., 2020). Hippo signaling compounds could regulate Notch pathways. In pancreatic cancer, curcumin reduces the expression of Yes-associated protein and transcriptional coactivator with PDZ-binding motif, two paralogous proteins belonging to the Hippo signaling pathway, which results in the subsequent suppression of Notch1 expression (Zhou et al., 2016). Kim et al. (2014) reported that overexpression of Notch1 prevented silybin-induced inhibition of ERK and AKT phosphorylation. We summarized the cross talk between Notch and other signaling pathways in different cancers with phytochemicals in Table 2.

**TABLE 2:** Phytochemicals on Notch and other pathways crosstalk.

Phytochemical	Cancer type	Cross talk	Reference
EGCG	Neuroblastoma	Notch, AKT, VEGF	Hossain et al. (2012)
	Liver cancer	Notch, Hedgehog, NF-κB	Ashry et al. (2022)
Resveratrol	Cervical cancer	Notch, STAT3, Wnt	Zhang et al. (2014)
	Ovarian cancer	Notch, STAT3, Wnt	Zhong et al. (2015)
	T-ALL	Notch, PI3K/AKT	Cecchinato et al. (2007)
	Ovarian cancer	Notch, PTEN, AKT	Kim et al. (2019)
Emodin	Glioma stem cells	Notch, Wnt, STAT3	Kim et al. (2015)
	Glioma stem cells	EGFR, Notch, Wnt, STAT3	Kim et al. (2018)
Quercetin	Breast cancer	Notch, PI3K/AKT	Cao et al. (2018)
	Breast cancer	Notch, Death-associated factor 6	Takebe et al. (2014)
	Histiocytic lymphoma	Notch, AKT, mTOR, VEGF	Chen et al. (2016b)
Curcumin	Oral cancer	Notch, NF-κB	Liao et al. (2011)
	Lung cancer	Notch, HIF1α, VEGF, NF-κB	Li et al. (2018)
	Pancreatic cancer	Notch, Hippo	Zhou et al. (2016)
Triptolide	Brain cancer	Notch, PI3K/AKT	Zhang et al. (2018)
Triptonide	Gastric cancer	Notch, NF-κB	Xiang et al. (2020)
Trichostatin A	Gastric cancer	Notch, NF-κB	Yao et al. (2012)
Genistein	Breast cancer	Notch, Death-associated factor 6	Peiffer et al. (2020)
	Pancreatic cancer	Notch, NF-κB	Wang et al. (2006c)
	Colon cancer	Notch, NF-κB, Ecadherin	Zhou et al. (2017)
	Pancreatic cancer	Notch, NF-κB	Wang et al. (2006b)
	Prostate cancer	Notch, AKT, foxm1	Wang et al. (2011a)
	Breast cancer	Notch, NF-κB	Pan et al. (2012)
	Pancreatic cancer	Notch, EMT	Wang et al. (2009)
	Pancreatic cancer	Notch, Cancer Stem Cells	Wang et al. (2011b)
Silybin	Breast cancer	Notch, AKT, ERK	Kim et al. (2014)
Berberine	Gastric cancer	Notch, MAPK, NF-κB	Wang et al. (2021)
Withaferin A	T-ALL	Notch, eIF2A	Sanchez-Martin et al. (2017)
	Colon cancer	Notch, JNK	Koduru et al. (2010)
	Colon cancer	Notch, AKT, Bcl-2	Koduru et al. (2009)
	Ovarian cancer	Notch, AKT, Bcl-2	Zhang et al. (2012)
Physciosporin	Colorectal cancer	Notch, Hedgehog	Yang et al. (2019)
(-)-Gossypol	Glioma stem cells	Notch, Hedgehog	Linder et al. (2019)
Cordycepin	Breast cancer	Notch, Hedgehog	Liu et al. (2020)
Esc-3	Ovarian cancer	Notch, Wnt	Fu et al. (2017)
8,12-Dimethoxysanguinarine	Breast cancer	Notch, NF-κB, PI3K/AKT, Wnt	Yang et al. (2023)
Okadaic Acid	Breast cancer	Notch, PI3K/AKT, NF-κB	Li et al. (2016)
Adriamycin Or Paclitaxel	Breast cancer	Notch, VEGF	Zhang et al. (2016)
Nimbolide	Oral cancer	Notch, VEGF, MMP	Kowshik et al. (2017)
Artemisinin	Breast cancer	Notch, VEGF, HIF1α	Dong et al. (2020)
Z-Ajoene	Glioblastoma multiforme	Notch, AKT, TGFβ	Jung et al. (2014)

## 4 Conclusion and perspective

Recently, there has been an increasing attraction in developing clinically potent antagonists of the Notch signaling pathway. Antitumor activity has been observed from gamma-secretase inhibitors and monoclonal antibodies administered as single agents in early clinical trials (Takebe et al., 2014). However, achieving Notch-directed therapeutics remains an unattained goal. Currently, no compounds, including chemicals and phytochemicals, that interfere with Notch signaling have been approved for using in cancer patients. Additionally, it is imperative to develop competent tools and precise research techniques to understand the heterogeneity and complexity of the Notch signaling pathway in cancer.

The research summarized above has shown that Notch activation and interactions with other oncogenic pathways strongly promote the malignant phenotype, *in vitro* and *in vivo*. To some extent, natural bioactive compounds can inhibit Notch activation and cross talk with other oncogenic pathways without any significant side effects. Further research is needed to elucidate the significance and mechanisms of Notch pathway activation in cancer, and to determine whether Notch-based therapies in combination with chemotherapy or other biologically targeted drugs could enhance clinical anti-tumor efficacy. And whether there is a need to discover more sophisticated and potent Notch inhibitors. Targeting Notch is critical because the pathway communicates extensively with other signaling pathways, such as Wnt, Hedgehog signaling, growth factors such as EGFR, TGF-β and VEGF, oncogenic kinases, and transcription factors such as NF-κB, PI3K/AKT/mTOR. Therefore, the multi-target properties of natural products and drugs designed to inhibit Notch oncogenic signaling crosstalk may have potential for therapeutic intervention.

In addition, an important future direction for Notch targeting is to ascertain the functions of the Notch pathway in various cancer cells and to develop a biomarker for cancer and stromal sensitivity.

## Abbreviations

Notch1/2/3/4, Notch receptor 1/2/3/4; DLL1/3/4, Delta-like protein 1/3/4; ADAM, A disintegrin and metalloprotease; NECD, extracellular domain; NICD, intracellular domain of Notch; CSL, CBF1, Suppressor of Hairless, Lag-1; EGCG, Epigallocatechin-3-gallate; GSIs, gamma-secretase inhibitors; RBPJ, recombination signal-binding protein of immunoglobulin kappa J region; PTCH, patched receptor; SMO, smoothened; SUFU, suppressor of fused; COS2, costal 2; PKA, protein kinase A; SLIMB, supernumerary limbs; Ci/Gli, Cubitus interruptus/zinc finger protein-Glioma-associated oncogene homolog1; ErbB2, HER2/Neu; PDGF, platelet-derived growth factor; EGFR, epidermal growth factor receptor; TGF-β, transforming growth factor type beta; PTEN, phosphatase and tensin homolog; PP2A, protein phosphatase 2; TCF/LEF, T-cell factor/lymphoid enhancer factor; PI3K, Phosphatidylinositol 3-Kinase; AKT, protein kinase B; ERK, extracellular signal-regulated kinase; mTOR, mammalian target of rapamycin.
